# Pattern of paediatric corneal laceration injuries in the University of port Harcourt teaching hospital, Rivers state, Nigeria

**DOI:** 10.1186/1756-0500-5-683

**Published:** 2012-12-13

**Authors:** Adio Adedayo Omobolanle, Nwachukwu Henrietta

**Affiliations:** 1 Department of Ophthalmology, University of Port Harcourt, Choba, Rivers state, Nigeria; 2 Eye clinic, University of Port Harcourt teaching hospital, Port Harcourt, Rivers state, Nigeria

**Keywords:** Corneal laceration, Missiles, Penetrating injury, Paediatric trauma, Ocular injury, University of port harcourt teaching hospital, Nigeria

## Abstract

**Background:**

Corneal lacerations mostly affect younger children, commonly males, who will constitute the majority of the workforce. Clinical outcomes are reviewed and compared so that measures to reduce their occurrence and improve outcome can be proffered.

**Methods:**

Records of all children between the ages of 1-18 yrs, who presented with penetrating eye injuries at the eye clinic of the University of Port Harcourt teaching Hospital, Rivers state, Nigeria between January 2002 and December 2009 were included. Information retrieved -patient’s Bio data, presenting symptoms, presenting visual acuity (VA), source of injury, surgical intervention and outcome using VA. All data analysed with EPI Info version 6 with the aid of a statistician.

**Results:**

Folders of thirty-six children (36 eyes) between the ages of 0–18 years diagnosed with corneal laceration over a period of 8 years out of 65 cases managed within that period available. Other folders reported as missing. Male female ratio 3:1, the mean age is 8.7 years (SD ± 3.67). Only one presented within 24 hours. Objects causing injury mainly missiles with stones/catapult injuries (n = 8, 22.2%). Presenting VAs in those that could be measured, ranged from 6/24 to 6/60 (n = 4, 11%) to no light perception (NLP) (n = 5, 13.9%). Associated injuries include lid laceration, cataract, vitreous haemorrhage and retinal detachment. Twenty one patients had primary corneal repair (58.3%) carried out within 7 days of presentation. Four had endophthalmitis. After 3 months follow up, VA of 6/60 and better was achieved in 11 of 18 eyes left in follow up (6/60-6/24 in 8 eyes (22.2%), 6/18 and better in 3 eyes (8.3%).

**Conclusion:**

Most eye injuries in children are preventable. In this study, the prognosis was better in those whose injuries were confined to a peripheral part of the cornea, with no other associated injury, who presented within 5 days and who did not have any intraocular infection at the time of presentation. The importance of health education, adult supervision of play and application of appropriate measures that is necessary for reducing the incidence and severity of trauma is emphasized.

## Background

A corneal laceration is a partial or full thickness injury to the cornea [1] and can result from direct trauma to the cornea typically from a metallic or other object impacting to breach the cornea layers with sufficient force [2].

Patients usually have an intensely painful eye (which may thereafter diminish slightly due to corneal desensitization) and they often present with a profusely lacrimating and photophobic eye.

In partial thickness injury, the intraocular pressure may drop to between 2-6 mmHg while in full thickness injury, aqueous humour escapes from the anterior chamber and this results in a flat appearance of the cornea with intraocular pressure which may not be recordable. There may be air bubbles under the cornea or an asymmetric pupil secondary to iris protruding through the corneal defect. There is usually significantly reduced visual acuity. There may be associated injury which may include lens dislocation and cataract formation, iridodialysis and hyphaema or posterior segment injuries like vitreous haemorrhage or retinal detachment depending on the severity of the injury [2].

Penetrating injuries of the cornea mostly affect younger people who are still active and constitute the majority of the workforce. This generally leads to the development of a corneal opacity which is a significant cause of blindness the world over, more in developing countries [3,4]. A retrospective study carried out in Ibadan showed children were affected in about a quarter of the penetrating injuries that they reviewed [5]. Currently, in the University of Port Harcourt teaching hospital, Rivers state, no study is available detailing the clinical outcome of these types of injuries in children through which our experiences can be compared with that occurring in other areas and as such, the mechanisms by which these injuries happen, the effects on the eyeball and the outcome of the treatment modalities particularly in our own peculiar environment are important and need to be studied so that measures to reduce their occurrence and improve outcome can be proffered and put in place by relevant authorities.

## Methods

Folders of all children between the ages of 1-18 yrs, who presented with corneal laceration at the eye clinic of the University of Port Harcourt teaching Hospital, Rivers state, Nigeria between January 2002 and December 2009, an 8 year period, were included in this study.

This eye care center, located in the outskirts is one of 2 eye care centers within the state capital of Port Harcourt, the other being state owned with some similarly diagnosed patients probably presenting there. The research was approved by the University of Port Harcourt teaching Hospital Ethical Board and conforms to the generally accepted principles outlined in the Helsinki declaration of 1964.

From the outpatient log book, 65 children folders were recorded but only 36 folders were retrievable from the Records department of the hospital. Reasons given include loss or misplacement during movement to another storage facility within the hospital. For the study we defined corneal laceration as a full thickness injury which penetrates completely through the cornea [2].

Information retrieved from available folders include the patient’s Bio data, presenting symptoms, presenting visual acuity, source of injury, surgical intervention and outcome as measured by the visual acuity with or without medical or surgical intervention. All data was analysed with EPI Info version 6 with the aid of a statistician.

## Results

Folders of thirty-six children with 36 eyes between the ages of 0–18 years who were diagnosed with corneal laceration over a period of 8 years were retrievable out of 65 eyes of 65 children that were seen during that period. This gave 55.4% retrieval rate.

Of the 36 patients that were available for study, twenty-six were males [72.2%] and ten were females [27.8%] male female ratio approximately 3 :1, the mean age is 8.7 years (SD ± 3.67). Their ages ranged from 6 months to 17 years See Table 1. 63.9% (n = 23) of injuries happened to children less than 10 years old.

**Table 1:** Age and sex distribution of paediatric patients with corneal laceration in UPTH

**Age Range (yrs)**	**Sex**		**Total**
	**Male**	**Female**	
	**Freq (%)**	**Freq (%)**	
**0-5**	12 (33.3)	3 (8.3)	15 (41.6)
**6-10**	6 (16.7)	3 (8.3)	8 (25.0)
**11-15**	7 (19.4)	2 (5.6)	11 (25.0)
**15 and Above**	1 (2.8)	2 (5.6)	2 (8.4)
**Total**	26 (72.2)	10 (27.8)	36 (100.0)

### Objects causing injury

Mainly missiles with stones/catapult injuries accounting for most cases (n = 8, 22.2%), then stick/broomstick injuries (6 cases, 16.7%) followed by broken bottle/exploded bottle top injuries (5 cases, 13.9%). Others are as detailed in Table 2. Majority occurred while playing either at home (n = 25, 69.4%) or at school (n = 11, 30.6%).

**Table 2:** Causes of corneal laceration among paediatric patients in UPTH

**Object causing injury**	**Number of cases**		**Total**
	**Male**	**Female**	
**Stones/Catapult**	7	1	8 (22.2)
**Broken bottles/bottle top**	2	3	5 (13.9)
**Stick/broom stick**	5	1	6 (16.7)
**Scissors/nail/screwdriver**	3	1	4 (11.0)
**Zinc roofing sheets/cutlass**	2	-	2 (5.6)
**Umbrella spoke**	2	-	2 (5.6)
**Dog bite**	1	-	1 (2.8)
**Not Known/documented**	4	4	8 (22.2)
**Total**	26	10	36 (100.0)

### Timing of presentation

Only one presented within 24 hours. By 48 hours, total of seven of the children had presented (19.4%). Most of the children presented within the first seven days (n = 15, 41.7%), 8 presented after one week (22.2%) See Table 3.

**Table 3:** Time of presentation of paediatric corneal lacerations in UPTH

**Time of presentation**	**Number**	**%**
**Within 24 hours**	1	2.8%
**Within 48 hours**	6	16.7%
**Between 24 hr and 1 week**	15	41.6%
**More than 1 week**	8	22.2%
**More than 1 month**	6	16.7%

### Presenting visual acuities

Presenting visual acuities in those that could be measured, ranged from 6/4 to 6/18(n = 3,8.4%), 6/24 to 6/60 (n = 4, 11%) while it was either between 3/60 and 1/60(n = 2, 5.6%),hand movement(HM)(n = 5,13.9%) light perception (LP) (n = 4, 11.1%) or no light perception (NLP) (n = 5, 13.9%) in others See Table 4.

**Table 4:** Presenting and postoperative visual acuities of paediatric patients with corneal laceration in UPTH

**Visual acuity range**	**Presenting visual acuities**	**Corrected postoperative visual acuities (4 weeks)**	**Corrected postoperative visual acuities (12 weeks)**
	**Freq%**	**Freq%**	**Freq%**
**6/4 to 6/18**	**3 (8.3)**	**8 (22.2)**	**3 (8.3)**
**6/24 to 6/60**	**4 (11.1)**	**4 (11.1)**	**8 (22.2)**
**3/60 to 1/60**	**2 (5.6)**	**3 (8.3)**	**-**
**HM**	**5 (13.9)**	**2 (5.6)**	**3 (8.3)**
**LP**	**4 (11.1)**	**1 (2.8)**	**2 (5.6)**
**NLP**	**8 (22.2)**	**8 (22.2)**	**2 (5.6)**
**Could not be taken**	**10 (27.8)**	**10 (27.8)**	**-**
**Lost to followup**	**-**	**-**	**18 (50.0)**
**Total**	**36 (100.0)**	**36 (100.0)**	**36 (100.0)**

### Associated ocular injuries

These include lid laceration (n = 1, 2.8%), traumatic cataracts (n = 8, 22.2%), vitreous haemorrhage (19.4%) and retinal detachment (5.6%). The patient with lid laceration was repaired primarily while the patients with vitreous haemorrhage and retinal detachment were referred for care by a vitreoretinal surgeon in a regional center. In this study, corneal lacerations affected both right and left eyes equally with uveal prolapse occurring in about sixteen children (44.4%) (See Table 5).

**Table 5:** Types of paediatric corneoscleral injuries presenting at UPTH

**Type of laceration**	**Freq%**
**Corneal injury alone**	9 (25.0)
**Corneal injury + uveal prolapse**	16 (44.4)
**Limbal involvement alone**	1 (2.8)
**Limbal involvement in addition**	20 (58.3)
**Scleral laceration alone**	1 (2.8)
**Corneoscleral laceration + complications**	9 (25.0)
**-**lid laceration---1 case	
-Cataract---4 cases	
-posterior segment(Vitreous haemorrhage, retinal detachment, endophthalmitis---4 cases.	

The limbus was involved in 21 cases (58.3%) See Table 6. Postoperative visual recovery was poorer due to associated complications and injuries to contiguous structures ‘like the lens and posterior segment occurring more often in this group. Associated cataracts that resulted from the trauma were extracted in 2 eyes concurrently with the repair but one did not have IOL insertion primarily due to young age (6 months old). Traumatic cataract extraction as a second stage operation was not done due to loss to follow up. Four cases had endophthalmitis (these were injured with a stick, 2 cases and broomstick, 2 cases). These were treated with topical, subconjunctival and intravenous antibiotics along with antifungals. Intravitreal antibiotics were advised but actually given in 1 case of broomstick injury 5 days after. It did not make any appreciable difference in visual recovery however in that one case however. The other cases could not be given due to inability to provide the drugs or discharge against medical advice See Tables 5 and 7 for other details.

**Table 6:** Timing of reparative surgery for corneoscleral laceration at UPTH

**Time of repair of corneal laceration**	**Freq%**
**Within 24 hours of presentation**	1 (2.8%)
**Within 7 days of presentation**	20 (55.6%)
**Repair > 7 days of presentation**	4 (11.1)
**Repair not done at all**	719.4%
**Had repair-date not presented**	4 (11.1)
**Total**	36 (100.0)

**Table 7:** Four week summary of treatment outcome of paediatric corneoscleral lacerations at UPTH

**4 week outcome after treatment of injury**	**Freq%**
**Vision restored to >6/18**	8 (22.2)
**Corneal scar requiring referral**	21 (58.3)
**Cataract requiring treatment**	4 (11.1)
**Overwhelming infection**	4 (11.1)
**Significant refractive errors**	12 (33.3)
**Referred for treatment of posterior segment injuries**	2 (5.6)
**no treatment taken**	7 (19.4)
**Irreversible blindness**	11 (30.6)

### Timing of surgical intervention

Twenty-one of the patients had a repair carried out within 1 week of presentation. Other details are as in Table 6.

For those presenting later than 4 weeks post injury(6 cases), surgery was not done as they were already healed and complicated with corneal opacities, anterior chamber granuloma formation and lens opacities with adherent leucomas which needed referral to a corneal transplant/anterior segment specialist.

The one patient that presented within 24 hours of injury (5 hours) sustained accidental injury from a cutlass with immediate loss of vision. Visual acuity on admission was NLP with extensive corneoscleral laceration with limbal involvement in association with uveal prolapse seen on examination. A repair was carried out but with no improvement in vision See Table 8.

**Table 8:** The presenting VA and outcome of paediatric patients who presented within 48 hours of injury at UPTH

**Nature of injury**	**Type of injury**	**Presenting visual acuity**	**Visual acuity on discharge**
**Zinc roofing sheets/cutlass (1 patient presented within 48 hr.)**	**Scleral laceration + uveal prolapse**	**CF**	**CF**
**Stick/broomstick (1 patient presented within 24 hr.)**	**Corneoscleral lac + uveal prolapse**	**NLP**	**NLP**
Broken bottles/bottle top(1 patient presented in 48 hr)	**Cornel laceration + uveal prolapse**	**NLP**	**NLP**
**Stone/catapult (4 patients presented within 48 hr.)-**	**Corneal laceration + uveal prolapse**	**CF**	**6/12**
**Patient 1**			
Patient 2	**Corneal laceration + uveal prolapse**	**HM**	**6/9**
**Patient 3**	**Corneoscleral lac + limbal involvement + uveal prolapse**	**NLP**	**NLP**
Patient 4	**Corneal laceration only**	**6/60**	**6/36**

### Outcome after 4 weeks

Overall only 8 of them had fair visual outcome (22.2%) (i.e. 6/18 or better) because of the severity of trauma mostly affecting the central part of the cornea and associated ocular injuries after 4 weeks of treatment See Table 4. About 7 patients required spectacle correction (19.4%). See Table 7 for other details about outcome after 4 weeks of treatment following injury.

### Outcome after 12 weeks

The maximum length of follow up achieved was 3 months in 18 patients (50%) and about 18 were already lost to follow up by then and so data was not complete for this period.

At the end of 3 months follow up, visual acuity achieved in 18 eyes are as follows: NLP in 2 eyes(5.6%) and 6/18 and better in 3 eyes (8.3%) See Table 4 for other details.

## Discussion

Penetrating injury is the leading cause of unilateral visual loss in children [6]. This was also the case in this study where 65% of injuries affected those less than 10 years old and causing severe loss of vision in majority of them. This was also the prevalent finding in an Ibadan study [4].

Most of the patients in our series were males (72.2%) This is similar to studies all over the world where the male sex has been found to be more prone to ocular injuries ranging from 63% to 77.4% [3-5,7-13]. Penetrating injuries involving the cornea usually lead to the development of corneal opacities. It is the third most common cause of blindness after cataract and glaucoma. In our study, this type of injury led to significant corneal opacities that required referral for a transplant (up to 21 cases). As donor corneas and cornea specialists are very few in number in this country, they usually remain blind in that eye.

In the Ashaye et al. (2004) study [4], trauma was the cause of corneal opacity in over half of the cases that presented. Nwosu (1998) also found out the same in his study in south eastern Nigeria [14]. This underscores the public health importance of ocular trauma as a significant cause of cornea blindness globally particularly in the developing countries. In our mainly agricultural setting, significant number of blinding injuries occurs from sticks, cutlass and related objects as shown in Table 2 detailing causes of injury.

In most of the cases where VA could be measured (others being too young to cooperate), patients presented with very poor vision in the blindness category (VA < than 3/60) with up to 25% in the LP and NLP range Table 4. This also agrees with Ashaye et al. (2004) [4] and Nwosu(1998) [14] studies. This is also comparable to a Middle Eastern study where the presenting VA was very poor in two thirds of the cases [10].

The prognosis after a penetrating injury is strongly influenced by the nature of the injury and the extent of initial damage. Other factors that may possibly correlate with an unfavourable visual outcome are;-nature of the object used, initial preoperative visual acuity, wounds involving the limbus and sclera, severe associated injuries e g lens opacity (significant enough in some of the cases to warrant an extraction), vitreous haemorrhage, retinal detachment (following which the visual outcome was poor in our series with some developing phthisis bulbi). Other factors include the sex of the patient (here shown to be a significant risk factor to sustaining this type of injuries) late surgical intervention (as in some of the cases in our series where surgery was not carried out immediately due to finances) and infection (all cases in our series that developed endophthalmitis lost their vision).

Most of the objects used in our series were missiles such as stones, and sticks. Other studies have documented the use of stones and sticks (wood) as the object of injury [7,9,15]. Mselle documented stones, sticks and metallic objects were the commoner causes of injury to the eye in his series [16]. The introduction of sticks into the eye raises the possibility of fungal infections developing which tend to linger.

Dog bite was the cause in one of our patients. This is unusual and has not been reported in literature yet as far as known. This however happened to a 6 month old infant who could not protect himself from attack by a dog. Bow and arrows were reported in an Indian study as the cause of injury [8]. This has not yet been reported in our locality as a cause in children. In our study, a significant number were injured during play at home. This was also the finding in other studies [8,9,15,17].

The wound was involving the limbus and sclera in 21 cases in our series (58.3%). This is similar to the Ibadan study where 65% of the subjects had a similar occurrence [5]. Wounds like this tend to have increased morbidity, difficult to manage astigmatic errors and more often than not have other associated injuries in the eye [2].

Associated injuries were noted in about half with some cases presenting with traumatic cataracts and vitreous haemorrhage. This was more common in the stick and broken glass injuries group. The outcome was worse if there was already an endophthalmitis as was the case in 4 eyes, all of whom had a visual acuity after treatment of either LP or NLP. Preoperative VA in these 4 cases was in the CF to LP group. These 4 cases gave an incidence of 11.1%. This is much higher than was published in an Iranian study that showed an incidence of 5.1% [18].

Visual recovery after treatment for those who consented was however encouraging as about half of 18 patients left in follow up by 3 months retained useful vision, ranging from 6/12 to 6/60 with the majority (n = 8, 22.2%) having visual acuity of between 6/24 to 6/60. Only 3 however had VA of 6/18 or better (8.3%). This is similar to a study carried out in Ibadan, Nigeria where the visual recovery in most cases was poor (only 19.3% of 60 children had VA of 6/12 or better postoperative) following penetrating eye injuries in children [5]. This was also demonstrated in other countries in the developing world [8]. In contrast to this, a Scottish study showed that the prognosis or outcome of ocular injuries has significantly improved with the vast majority in their area having an excellent visual outcome [19]. This is not out of place with the present trend worldwide where corneal related injuries are more common in the developing world than in the developed [3]. Also corneal transplant services may be more frequently available there than here in the developing world. There were slightly better results at 4 weeks postoperative however. These figures may however be biased as half of the people were later lost to follow up and their own outcome may have influenced the final results.

The diagnosis of corneal laceration must be made as quickly as possible with as little unskilled intervention or manipulation as possible therefore it is highly important for the patient to present early to afford better visual outcomes. In this study however, most patients presented late and this significantly affected outcome. Only one person presented within 24 hours after the injury. This is similar to studies carried out elsewhere [5,9]. This was probably due to financial constraints as they also had surgery late (also due to finance).

This directly affected outcome as some were already infected before presentation. Some may have resorted to self-medication or the use of harmful traditional eye medications (HTEM) prior to presentation as documented by in a Tanzanian study [16], where up to half of the patients were found to have used it. The visual outcome was worse among these Tanzanians prior to presentation following injuries to the eye with higher rates of keratitis, endophthalmitis and panophthalmitis. However a South African study reported up to 25% presenting within 24 hours of injury [15].

Despite these challenges, most (about 11 of 18) patients who had a minimum follow up of 3 months had at least a moderate visual outcome (Tables 4 and 7).

## Conclusion

Ocular trauma in children is associated with visual loss. In this study, the prognosis was better in those whose injuries were confined to a peripheral part of the cornea, those who had no other associated injury particularly posterior segment, those who presented within 5 days and those who did not have any intraocular infection at the time of presentation.

Many of the cases were preventable. Public education of care givers on preventive strategies and importance of supervised play in addition to early presentation, general awareness and aggressive primary management, possibly subsidized may be indicated to optimize visual outcome.

## Competing interests

The authors declare that there are no competing interests.

## Authors’ contributions

The author NH collected folders and all the data from the folders and wrote the introduction, results and discussion in part. AOA completed the introduction, methodology, results, illustrations and discussion and the conclusion and properly referenced the paper. Both authors read and approved the final manuscript.
